# Improving prenatal diagnosis with combined karyotyping, CNV-seq and QF-PCR: a comprehensive analysis of chromosomal abnormalities in high-risk pregnancies

**DOI:** 10.3389/fgene.2024.1517270

**Published:** 2025-01-13

**Authors:** Jia-pei Liu, Shan-Bing Wang, Li Luo, Ya-mei Guo

**Affiliations:** ^1^ Department of Laboratory, The Second People’s Hospital of Yibin City, Yibin, Sichuan, China; ^2^ Department of Oncology Medicine, The Second People’s Hospital of Yibin City, Yibin, Sichuan, China

**Keywords:** chromosomal abnormalities, prenatal diagnosis, karyotyping, CNV-seq, QF-PCR, high-risk pregnancy, copy number variations

## Abstract

**Objective:**

This study aims to assess the diagnostic efficacy of a combined approach integrating chromosomal karyotyping, copy number variation sequencing (CNV-seq), and quantitative fluorescence polymerase chain reaction (QF-PCR) in detecting chromosomal abnormalities in high-risk pregnancies.

**Methods:**

This retrospective study analyzed 617 high-risk pregnancies undergoing prenatal diagnosis from February 2023 to August 2024, with amniotic fluid samples concurrently analyzed using karyotyping, CNV-seq, and QF-PCR. We evaluated clinical characteristics, diagnostic yields, and inter-method concordance rates. Longitudinal follow-up assessed pregnancy outcomes and neonatal phenotypes, with particular emphasis on cases demonstrating diagnostic discrepancies or variants of uncertain clinical significance.

**Results:**

The integrated approach detected chromosomal abnormalities in 12.5% (77/617) of cases, significantly higher than the rates achieved by karyotyping alone (9.7%) and CNV-seq/QF-PCR alone (8.3%) (*p* < 0.05). Karyotyping showed full concordance with CNV-seq and QF-PCR in detecting major chromosomal aneuploidies, identifying 21 cases of trisomy 21 and 4 cases of trisomy 18. CNV-seq uniquely identified additional pathogenic copy number variations in 2.1% of cases and variants of uncertain significance (VUS) in 3.2% of cases, both undetectable by conventional karyotyping. Subjects with high-risk non-invasive prenatal testing (NIPT) results had the highest abnormality detection rate (57.6%, *p* < 0.05). Follow-up data revealed pregnancy termination in 44 of 97 cases with chromosomal abnormalities. Notably, neonates carrying pathogenic CNVs inherited from asymptomatic parents demonstrated normal phenotypes.

**Conclusion:**

The integration of karyotyping, CNV-seq, and QF-PCR provides superior diagnostic yield compared to individual testing strategies in high-risk pregnancies. Although karyotyping remains the gold standard for detecting major chromosomal aberrations, CNV-seq and QF-PCR enhance diagnostic precision through detection of submicroscopic variations. Multi-center studies with larger cohorts are needed to confirm these findings and clarify the clinical significance of uncertain variants.

## Introduction

In China, congenital anomalies occur at a prevalence of approximately 5.6% (Xu et al., 2020). These developmental defects result from aberrant fetal morphogenesis *in utero*, potentially affecting the fetus’s anatomy, physiology, and cognitive development (Zhou et al., 2024). Consequences range from intrauterine fetal demise to congenital malformations and neurodevelopmental disorders. Chromosomal abnormalities, such as aneuploidy, triploidy, and large-scale deletions or duplications, are the primary etiological factors (Kagan et al., 2022). Moreover, submicroscopic chromosomal aberrations and copy number variations (CNVs) contribute significantly to the pathogenesis of congenital anomalies (Cao et al., 2023; Chen et al., 2023). Currently, pregnancy termination following prenatal diagnosis of fetal abnormalities remains the predominant management strategy (Graf et al., 2023).

Amniotic fluid karyotyping has long been regarded as the gold standard for diagnosing chromosomal abnormalities. For 5 decades, this technique has been widely utilized to identify conditions such as aneuploidy, polyploidy, balanced structural rearrangements, and large chromosomal segment abnormalities (Luo et al., 2023). However, this method has several limitations: it requires amniotic fluid cell culture, requires skilled technicians, is time-consuming, and lacks the resolution to detect copy number variations smaller than 5 Mb (Saldarriaga et al., 2015).

To address these limitations, genome-wide copy number variation sequencing (CNV-seq) based on low-depth whole-genome sequencing, combined with quantitative fluorescent PCR (QF-PCR) targeting short tandem repeats and chromosomal microarray analysis (CMA), has been recommended in China for prenatal diagnosis (Clinical et al., 2019; Prenatal et al., 2023). These advanced methodologies offers high-throughput capabilities, rapid turnaround times, and the ability to identify small CNVs(Chen et al., 2024; Qiao et al., 2022; Cai et al., 2023; Santirocco et al., 2021). Notably, CNV-seq demonstrates higher sensitivity, improved resolution, and greater robustness with suboptimal specimens compared to CMA (Cohen et al., 2011; Cohen et al., 2015; Ma et al., 2021). Nevertheless, these methods cannot detect balanced chromosomal translocations (Qiao et al., 2022). Therefore, integrating karyotyping with CNV-seq and QF-PCR may provide a more comprehensive assessment of chromosomal abnormalities.

In this study retrospectively analyzes data from 617 cases that underwent combined testing, aiming to evaluate the clinical utility of integrating karyotyping, CNV-seq, and QF-PCR in prenatal diagnosis.

## Methods

### Study population

This retrospective study analyzed 617 high-risk pregnant women who underwent prenatal diagnostic testing at our institution between February 2023 and August 2024. Patients were eligible if they underwent amniocentesis with subsequent chromosomal karyotyping, copy number variation sequencing (CNV-seq), and quantitative fluorescence polymerase chain reaction (QF-PCR) testing. Indications for prenatal diagnosis included ultrasonographically detected fetal structural anomalies, elevated risk on non-invasive prenatal testing (NIPT), family history of genetic disorders, previous adverse pregnancy outcomes, or other high-risk factors. Cases with insufficient sample volume, contamination, or technical failure were excluded. All participants provided written informed consent prior to procedures. The study protocol was approved by our institutional ethics committee.

### Sample collection and processing

Amniocentesis of 20 mL were obtained from each patient, equally divided for conventional karyotyping (10 mL) and combined CNV-seq/QF-PCR analysis (10 mL). The latter analyses were conducted at West China Second Hospital. Sample processing and cell culture were performed in our institutional genetics laboratory following standardized sterile protocols.

### Chromosomal karyotyping

For karyotyping, the amniotic fluid samples were centrifuged (1800 rpm, 10 min), and the pelleted cells were resuspended in 3 mL culture medium. Cultures were maintained at 37°C with 5% CO₂ for 11–14 days. Following harvest, G-banded chromosome preparations were analyzed using standard protocols. Two certified cytogeneticists (>5 years experience) independently evaluated 20 metaphase spreads per case, with detailed structural analysis of five randomly selected spreads. Karyotypes were reported according to the International System for Human Cytogenomic Nomenclature (ISCN, 2020), with final review by a senior cytogeneticist (associate professor or higher rank).

### Molecular genetic analysis

#### CNV-seq analysis

Genomic DNA was extracted from 4-mL aliquots of amniotic fluid specimens using standardized extraction protocols. To minimize PCR-induced artifacts, we implemented a PCR-free library preparation methodology (Berry Genomics Co., Ltd.). DNA library quantification was performed using Qubit 1X dsDNA High Sensitivity and Broad Range assay kits (Thermo Fisher Scientific) to determine precise DNA concentrations. Sequencing was conducted on the NextSeq CN500 platform using 36-bp single-end reads at approximately 0.1x coverage depth. Sequence reads were aligned to the GRCh37 human reference genome using the BWA-MEM algorithm, which enables high-fidelity mapping of short-read sequences. Copy number variation (CNV) analysis was performed using a read-depth approach, wherein significant deviations from expected genomic coverage were identified as putative CNVs. All detected variants were filtered and classified according to the American College of Medical Genetics and Genomics (ACMG) guidelines into five categories: pathogenic, likely pathogenic, variants of uncertain significance (VUS), likely benign, and benign. Rigorous quality control measures were implemented throughout the workflow, encompassing library preparation, sequencing metrics, and variant interpretation to ensure robust detection of clinically relevant CNVs. The final diagnostic report included only pathogenic and likely pathogenic variants.

#### QF-PCR analysis

DNA extracted from 4 mL amniotic fluid (TIANamp Genomic DNA Kit) was quantified by Qubit analysis and appropriately diluted. PCR amplification targeted 20 polymorphic short tandem repeat (STR) loci across chromosomes 13, 18, 21, and sex chromosomes using the Bio-Rad PTC 200 system. Analyzed markers included D13S628, D13S742, D13S634, D13S305, D18S1002, D18S391, D18S535, D18S386, D21S1433, 21q11.2, D21S1411, D21S1414, D21S1412, D21S1445, AMXY, DXS1187, DXS8377, DXS6809, DXS981, and SRY. PCR products were analyzed using an ABI 3500 genetic analyzer and GeneMapper 5.0 software.

Results from both molecular analyses were interpreted using the GRCh37 genome build and multiple reference databases (DGV, ClinGen, DECIPHER, GeneReviews, OMIM, UCSC, PubMed, gnomAD, and ClinVar).

#### Follow-up and statistical analysis

Pregnancy outcomes were tracked through telephone follow-up or medical record review, with particular emphasis on cases showing discordant results between karyotype and CNV-seq analyses, or those with VUS findings. Statistical analysis employed SPSS version 19.0. Categorical variables were expressed as percentages and compared using chi-square tests; continuous variables were expressed as mean ± standard deviation and analyzed using t-tests. Statistical significance was defined as *P* < 0.05.

## Results

### Study population and clinical characteristics

This study analyzed 617 pregnant women with a mean age of 30.5 ± 6.5 years and mean gestational age of 20.1 ± 2.2 weeks. Among the participants (N = 617), the primary indications for prenatal diagnosis were abnormal Down syndrome screening (47.6%, n = 294), advanced maternal age (33.2%, n = 205), high-risk non-invasive prenatal testing (NIPT) (5.3%, n = 33), adverse obstetric history (4.2%, n = 26), fetal structural anomalies on ultrasound (3.4%, n = 21), family history of genetic disorders (1.3%, n = 8), and other risk factors (4.9%, n = 30) (Table 1).

**TABLE 1:** Clinical characteristics.

Characteristic	Study population (617)
Maternal age (years)	30.5 ± 6.5
<35	412 (66.8%)
≥35	205 (33.2%)
Nation	
Han	563 (91.2%)
Miao	54 (8.8%)
Gestational age (weeks)	20.1 ± 2.2
Indications for invasive prenatal diagnosis	
High risk of Down’s syndrome screening	294 (47.6%)
Advanced maternal age	205 (33.2%)
NIPT high-risk	33 (5.3%)
Dverse pregnancy history	26 (4.2%)
Abnormal B-ultrasound	21 (3.4%)
Family genetic history	8 (1.3%)
Other	30 (4.9%)

### Conventional karyotype analysis

Chromosomal abnormalities were identified in 60 cases (9.7%) through cytogenetic analysis of amniotic fluid samples. These comprised 19 cases of trisomy 21, 4 cases of trisomy 18, and 9 cases of sex chromosome anomalies. The sex chromosome abnormalities included four cases of 45,X/46,XX/XY mosaicism, and single cases of 47,XXX, 47,XYY, 47,XXX/45,X/46,XX mosaicism, 46,X,psu idic(X) (p11.2), and X duplication (dup(X) (q26q28)). Additional findings included one case of low-level mosaic trisomy 20 (47,XX,+20 [4]/46,XX [43]), two autosomal structural abnormalities (46,XY,del (18) (p11.2) and 46,XN,der (4)t (4; 10) (p15.3; q24)), two balanced translocations (46,XX,t (3; 18) (q26; q22) and 46,XY,der (13; 14) (q10; q10)), and 23 cases of chromosomal polymorphisms.

### Combined CNV-seq and QF-PCR analysis

The integrated CNV-seq and QF-PCR approach identified chromosomal abnormalities in 51 cases (8.3%). These included 19 cases of trisomy 21, 4 cases of trisomy 18, and 12 cases of sex chromosome abnormalities (4 cases of X/XX mosaicism, 2 cases of X/XY mosaicism, 1 case of XXX/X mosaicism, 1 case of XXY/XY mosaicism, 1 case of 47,XXX, 1 case of 47,XYY, 1 case of X duplication at Xq26.3q28, and 1 case of Xq duplication with partial deletion at Xq22.33p11.1). Additional findings included one case of low-level mosaic trisomy 12, 13 clinically significant pathogenic CNVs or gene mutations (Table 2), and 20 variants of uncertain significance (VUS). The combined molecular approach achieved a significantly higher detection rate (12.5%, 77/617) compared to either method alone (*p* < 0.05).

**TABLE 2:** Pathogenic information of CNVs detected by CNV-seq.

No.	Karyotype	CNVs	Fragment size of CNVs	Clinical manifestations
1	46,XX	sseq [GRCh37]22q11.21 (18,880,000–20300,000)×3	1.42 Mb (microduplications)	22q11 duplication syndrome
2	46,XX	sseq [GRCh37]15q11.2q13.3 (27,620,000–32,460,000)×3	4.84 Mb (microduplications)	Autism
3	46,XY	sseq [GRCh37]22q11.21 (18,880,000–20300,000)×3	1.42 Mb (microduplications)	22q11 duplication syndrome
3	46,XX	sseq [GRCh37]16p11.2 (29,640,000–30200,000)×1	0.56 Mb (microdeletions)	microdeletion syndrome
4	46,XX	sseq [GRCh37]18p11.32p11.21 (11,320,000–14,980,000)×1	3.66 Mb (microdeletions)	Facial dysmorphism
5	46,XX	sseq [GRCh37]2q11.1q11.2 (96200000–97680000)×1	1.48 Mb (microdeletions)	Intellectual disability
6	46,XX	sseq [GRCh37]22.q11.21 (18,880,000–21,460,000)×3	2.58 Mb (microduplications)	22q11 duplication syndrome
7	46,XX	sseq [GRCh37]Xq28 (153,560,000–153,840,000)x3	0.28 Mb (microduplications)	22q11 duplication syndrome
8	46,XY	sseq [GRCh37]16p11.2 (23,640,000–30200,000)x3	1.72 Mb (microduplications)	Developmental delay, autism
9	46,XY	sseq [GRCh37]22q11.21 (18,880,000–21,480,000)x3	2.6 Mb (microduplications)	22q11 duplication syndrome
10	46,XX	sseq [GRCh37]Xp22.31 (6,460,000–8140,000)x0	1.68 Mb	Kallmann syndrome
11	46,XY	sseq [GRCH37]22q11.21 (18,880,000–21,460,000)x1	2.58 Mb (microdeletions)	22q11 duplication syndrome
12	46,XX	sseq [GRCh37]Xq23q24 (114,480,000–119,180,000)x1	4.7 Mb (microdeletions)	Intellectual disability
13	46,XY	sseq [GRCh37]10q26.2q26.3 (129,760,000–135,440,000)×1	5.6 Mb (microdeletions)	Intellectual disability

### Concordance analysis

**FIGURE 1:**
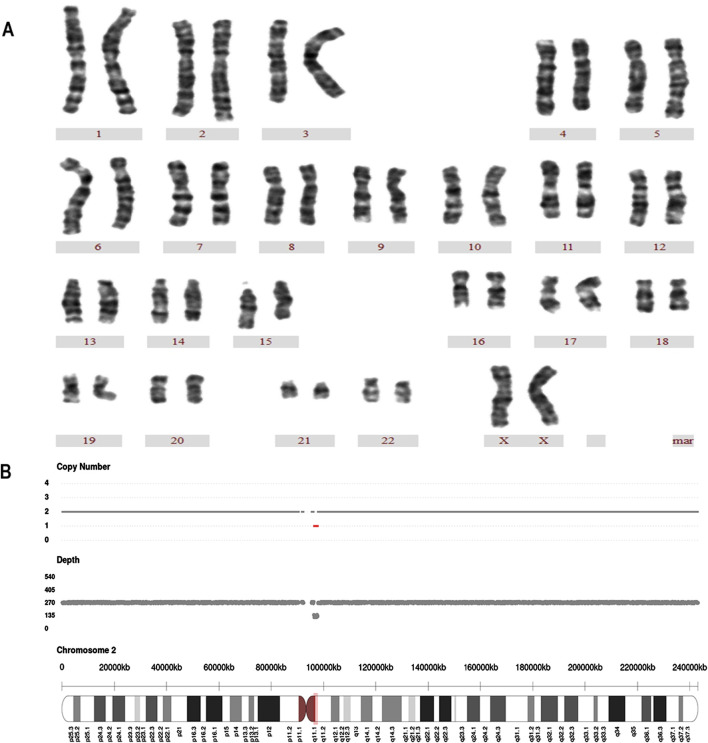
A case of Karyotyping and CNV-seq results **(A)** Karyotype analysis revealed a normal chromosomal structure. The karyotype was 46 XX. **(B)** CNV-seq was sseq [GRCh37]2q11.1q11.2 (96200000–97680000)×1, the 1.48 Mb region was deleted at q11.1-q11.2 on chromosome 2.

Karyotyping and combined CNV-seq/QF-PCR demonstrated 94.0% concordance (580/617 cases). Perfect concordance was observed for trisomy 21 and trisomy 18 detection. However, the molecular methods failed to detect chromosomal polymorphisms and balanced translocations identified by conventional karyotyping (Table 3). The results of karyotype analysis combined with CNV-seq and QF-PCR are presented in Figure 1.

**TABLE 3:** Comparison of numerical chromosomal abnormalities detected in the karyotyping, CNV-sep and QF-PCR groups.

	Karyotype	CNV-sep and QF-PCR
Trisomy 21	19	19
Trisomy 18	4	4
Sex chromosome aneuploid	9	12
Low-percentage trisomy 20 mosaicism	1	0
Autosomal structural abnormalities	2	2
Balanced translocations	2	0
Chromosomal polymorphisms	23	0
Low-percentage trisomy 13 mosaicism	0	1
Pathogenic CNVs	0	13
Variants of uncertain significance CNVs	0	20
Total	60	71

### Risk-stratified detection rates

Detection rates varied significantly among risk groups using the combined approach: high-risk NIPT (57.6%, 19/33), advanced maternal age (11.2%, 23/205), abnormal Down syndrome screening (9.5%, 28/294), family history (12.5%, 1/8), adverse obstetric history (11.5%, 3/26), other risk factors (6.7%, 2/30), and fetal ultrasound abnormalities (4.8%, 1/21). The high-risk NIPT group demonstrated significantly higher detection rates compared to all other groups (*p* < 0.05) (Table 4).

**TABLE 4:** Chromosomal abnormalities in pregnancies with different indications.

Indicators for prenatal diagnosis	Karyotype	CNV-sep and QF-PCR	Combined
High risk of Down’s syndrome screening	7.1% (21/294)	6.8% (19/294)	9.5% (28/294)
Advanced maternal age	9.2 (19/205)	5.4% (11/205)	11.2% (23/205)
NIPT high-risk	45.5% (15/33)	51.5% (17/33)	57.6% (19/33)
Dverse pregnancy history	3.8% (1/26)	7.7% (2/26)	11.5% (3/26)
Abnormal B-ultrasound	4.8% (1/21)	4.8% (1/21)	4.8% (1/21)
Family genetic history	12.5% (1/8)	12.5% (1/8)	12.5% (1/8)
Other	6.7% (2/30)	0% (0/30)	6.7% (2/30)

### Pregnancy outcomes and follow-up

Complete follow-up data were available for all 617 cases through 21 September 2024. Among the 97 cases with abnormal findings, 44 pregnancies were terminated, including cases of trisomy 21 (n = 19), trisomy 18 (n = 4), trisomy 20 (n = 1), sex chromosome abnormalities (n = 9), pathogenic CNVs (n = 10), and VUS (n = 1). Of the remaining cases, 482 resulted in live births, with 89 pregnancies ongoing at the time of analysis. Two live-born infants carrying maternally inherited pathogenic CNVs were healthy at birth. All 18 infants with VUS showed normal phenotypes postnatally. Inheritance patterns were established for 25 CNV cases: among pathogenic variants, 12 had confirmed parental origin (3 paternal, 7 maternal, 2 *de novo*), while VUS showed predominantly paternal inheritance (8 paternal, 4 maternal, 1 *de novo*).

## Discussion

Our integrated approach combining conventional karyotyping with CNV-seq and QF-PCR yielded a chromosomal abnormality detection rate of 12.5% (77/617), surpassing both isolated karyotyping (9.7% [60/617]) and CNV-seq/QF-PCR alone (8.3% [51/617]). This enhanced detection rate surpasses previously reported rates for CNV-seq as a standalone method and demonstrates comparable efficacy to the combined use of karyotyping and CNV-seq described in the literature (Wang et al., 2018; Zhang et al., 2022; Zhang et al., 2023; Ge et al., 2024).

Karyotyping remains the gold standard for chromosomal analysis, effectively detecting structural aberrations larger than 5–10 MB, aneuploidies, and balanced translocations. Our findings corroborate previous studies (Tang et al., 2024) demonstrating comparable efficacy between karyotyping and CNV-seq/QF-PCR in detecting major structural and numerical abnormalities, including trisomy 21 and 18. Notably, karyotyping identified chromosomal polymorphisms in 3.7% (23/617) of cases, two cases of balanced translocations, and one case of trisomy 20, underscoring its utility in detecting morphological chromosomal variants (Jing et al., 2021). While chromosomal polymorphisms and balanced translocations are not typically considered direct causative factors for congenital anomalies, emerging evidence suggests potential associations with subsequent infertility and oncological developments (Liehr, 2022; Pei et al., 2021; Ralapanawe et al., 2023; Verdoni et al., 2021). Thus, early identification of these variants provides valuable prognostic information for clinical surveillance and management strategies.

Our analysis detected CNVs in 5.3% (33/617) of cases, comprising 2.1% (13/617) pathogenic CNVs and 3.2% (20/617) variants of uncertain significance (VUS), consistent with published literature (Han et al., 2024; Xu et al., 2020; Zhang et al., 2022). These CNVs remained undetectable by conventional karyotyping. Notably, most CNV-carrying fetuses demonstrate no overt ultrasonographic abnormalities during gestation (Hilger et al., 2020; Jiang et al., 2024; Xue et al., 2021), underscoring the limitations of traditional prenatal screening methods and the value of incorporating molecular diagnostic techniques. However, the clinical interpretation of CNVs presents ongoing challenges. We observed two cases where fetuses carrying pathogenic CNVs (22q11.21 and 15q11.2 microduplications) exhibited normal phenotypes postnatally, with asymptomatic maternal carriers. These observations align with growing evidence of incomplete penetrance and variable expressivity in certain pathogenic CNVs(Hilger et al., 2020; Hu et al., 2017; Ramalingam et al., 2011), challenging the traditional paradigm of universal pregnancy termination for all pathogenic variants. Moreover, the detection of VUS poses significant counseling challenges, potentially inducing unwarranted anxiety and precipitous decision-making. In one instance, pregnancy termination was pursued due to family anxiety despite the absence of parental comparative analysis. This highlights the importance of exercising caution when CNVs are detected. Physicians and families should consider parental origin testing to inform decision-making and guide appropriate clinical management.

Although the mechanistic underpinnings and preferential genomic locations of CNVs remain not fully understood, our cohort showed a predominance of 22q11.21 microduplications (6/13) and 16p11.2 microduplications (2/13) among pathogenic CNVs. This distribution differs from previous reports by Zhang et al. (2023), who identified 15q11.2 microdeletions and 22q11.21 microduplications as the most prevalent pathogenic variants. VUS demonstrated no discernible pattern of occurrence. Intriguingly, we observed a preponderance of maternal inheritance for pathogenic CNVs, while VUS showed predominantly paternal inheritance. However, these findings warrant validation in larger cohorts.

Our data demonstrated that the detection rates of chromosomal abnormalities was was significantly higher in high-risk NIPT results (57.6%) compared to other risk categories, validating NIPT’s utility as a screening modality. Nevertheless, these findings reinforce that NIPT should not be employed as a diagnostic tool in isolation.

In conclusion, our findings validate the enhanced diagnostic yield of integrating karyotyping with CNV-seq and QF-PCR. However, the single-center, retrospective nature of this study necessitates further validation in larger, multi-center cohorts.

## Data Availability

The original contributions presented in the study are included in the article/Supplementary Material, further inquiries can be directed to the corresponding author.

## References

[B1] Cai M. Lin N. Guo N. Su L. Wu X. Xie X. (2023). Using single nucleotide polymorphism array for prenatal diagnosis in a large multicenter study in southern China. Sci. Rep. 13 (1), 7242. 10.1038/s41598-023-33668-0 37142625 PMC10160013

[B2] Cao L. Dong W. Wu Q. Huang X. Zeng X. Yang J. (2023). Advanced maternal age: copy number variations and pregnancy outcomes. Front. Genet. 14, 1206855. 10.3389/fgene.2023.1206855 37396033 PMC10308028

[B3] Chen Q. Zhang H. Li X. Li J. Chen H. Liu L. (2023). Sequential application of copy number variation sequencing and quantitative fluorescence polymerase chain reaction in genetic analysis of miscarriage and stillbirth. Mol. Genet. Genom. Med. 11 (8), e2187. 10.1002/mgg3.2187 PMC10422063 37073418

[B4] Chen Y. Han X. Hua R. Li N. Zhang L. Hu W. (2024). Copy number variation sequencing for the products of conception: what is the optimal testing strategy. Clin. Chim. Acta. 557, 117884. 10.1016/j.cca.2024.117884 38522821

[B5] Clinical G. G. O. M. Professional C. F. P. D. Group O. G. D. P. (2019). Expert consensus on the application of low-depth whole genome sequencing in prenatal diagnosis. Zhonghua Yi Xue Yi Chuan Xue Za Zhi 36 (4), 293–296. 10.3760/cma.j.issn.1003-9406.2019.04.001 30950010

[B6] Cohen K. Tzika A. Wood H. Berri S. Roberts P. Mason G. (2015). Diagnosis of fetal submicroscopic chromosomal abnormalities in failed array cgh samples: copy number by sequencing as an alternative to microarrays for invasive fetal testing. Ultrasound Obstet. Gynecol. 45 (4), 394–401. 10.1002/uog.14767 25510919

[B7] Cohen K. E. M. J. Morgan J. Wood H. Tsika A. Berri S. Mason G. C. (2011). Molecular karyotyping using massively parallel sequencing – a next generation approach to prenatal diagnosis? Arch. Dis. Child.-Fetal Neonatal Ed. Suppl. 1 (96), a54. 10.1136/adc.2011.300161.3

[B8] Ge Y. Chen J. Huang Y. Shao D. Wang W. Cai M. (2024). Retrospective study revealed integration of cnv-seq and karyotype analysis is an effective strategy for prenatal diagnosis of chromosomal abnormalities. Front. Genet. 15, 1387724. 10.3389/fgene.2024.1387724 38846960 PMC11153659

[B9] Graf W. D. Cohen B. H. Kalsner L. Pearl P. L. Sarnat H. B. Epstein L. G. (2023). Fetal anomaly diagnosis and termination of pregnancy. Dev. Med. Child. Neurol. 65 (7), 900–907. 10.1111/dmcn.15528 36732680

[B10] Han K. Yifei C. Lingxi W. Chonglan G. Xingyu L. Yu H. (2024). Pathogenic recurrent copy number variants in 7,078 pregnancies via chromosomal microarray analysis. J. Perinat. Med. 52 (2), 171–180. 10.1515/jpm-2022-0580 38081620

[B11] Hilger A. C. Dworschak G. C. Reutter H. M. (2020). Lessons learned from cnv analysis of major birth defects. Int. J. Mol. Sci. 21 (21), 8247. [Journal Article; Review]. 10.3390/ijms21218247 33153233 PMC7663563

[B12] Hu P. Wang Y. Sun R. Cao L. Chen X. Liu C. (2017). Copy number variations with isolated fetal ventriculomegaly. Curr. Mol. Med. 17 (2), 133–139. 10.2174/1566524017666170303125529 28260505

[B13] Jiang X. Liang B. He S. Wu X. Zhao W. Xue H. (2024). Prenatal diagnosis and genetic study of 22q11.2 microduplication in Chinese fetuses: a series of 31 cases and literature review. Mol. Genet. Genom. Med. 12 (7), e2498. [Journal Article; Review]. 10.1002/mgg3.2498 PMC11258554 39031005

[B14] Jing X. Liu H. Zhu Q. Liu S. Liu J. Bai T. (2021). Clinical selection of prenatal diagnostic techniques following positive noninvasive prenatal screening results in southwest China. Front. Genet. 12, 811414. 10.3389/fgene.2021.811414 35154255 PMC8834880

[B15] Kagan K. O. Sonek J. Kozlowski P. (2022). Antenatal screening for chromosomal abnormalities. Arch. Gynecol. Obstet. 305 (4), 825–835. 10.1007/s00404-022-06477-5 35279726 PMC8967741

[B16] Liehr T. (2022). Chromosomal heteromorphisms and cancer susceptibility revisited. Cells 11 (20), 3239. [Journal Article; Review]. 10.3390/cells11203239 36291106 PMC9600968

[B17] Luo H. Wang Q. Fu D. Gao J. Lu D. (2023). Additional diagnostic value of CNV-seq over conventional karyotyping in prenatal diagnosis: a systematic review and meta-analysis. J. Obstet. Gynaecol. Res. 49 (7), 1641–1650. 10.1111/jog.15652 37037422

[B18] Ma N. Xi H. Chen J. Peng Y. Jia Z. Yang S. (2021). Integrated cnv-seq, karyotyping and snp-array analyses for effective prenatal diagnosis of chromosomal mosaicism. BMC Med. Genomics. 14 (1), 56. 10.1186/s12920-021-00899-x 33632221 PMC7905897

[B19] Pei Z. Deng K. Lei C. Du D. Yu G. Sun X. (2021). Identifying balanced chromosomal translocations in human embryos by oxford nanopore sequencing and breakpoints region analysis. Front. Genet. 12, 810900. 10.3389/fgene.2021.810900 35116057 PMC8804325

[B20] Prenatal S. A. D. G. Prenatal D. G. S. O. Liu J. (2023). Guidelines for the application of chromosomal microarray analysis in prenatal diagnosis (2023). Zhonghua Yi Xue Yi Chuan Xue Za Zhi 40 (9), 1051–1061. 10.3760/cma.j.cn112141-20230327-00146 37643949

[B21] Qiao J. Yuan J. Hu W. Li Q. Fang H. Xu Y. (2022). Combined diagnosis of qf-pcr and cnv-seq in fetal chromosomal abnormalities: a new perspective on prenatal diagnosis. J. Clin. Lab. Anal. 36 (4), e24311. 10.1002/jcla.24311 35195919 PMC8993611

[B22] Ralapanawe M. Khattak H. Hapangama H. R. Weerakkody G. R. Papadopoulou A. Gallos I. (2023). Chromosomal polymorphisms in assisted reproduction: a systematic review and meta-analysis. Hum. Fertil. 26 (3), 687–698. 10.1080/14647273.2022.2051614 35322731

[B23] Ramalingam A. Zhou X. G. Fiedler S. D. Brawner S. J. Joyce J. M. Liu H. Y. (2011). 16p13.11 duplication is a risk factor for a wide spectrum of neuropsychiatric disorders. J. Hum. Genet. 56 (7), 541–544. 10.1038/jhg.2011.42 21614007

[B24] Saldarriaga W. Garcia-Perdomo H. A. Arango-Pineda J. Fonseca J. (2015). Karyotype versus genomic hybridization for the prenatal diagnosis of chromosomal abnormalities: a metaanalysis. [Comparative study; journal article; meta-analysis; review; systematic review]. Am. J. Obstet. Gynecol. 212 (3), 330–331. 10.1016/j.ajog.2014.10.011 25305409

[B25] Santirocco M. Plaja A. Rodo C. Valenzuela I. Arevalo S. Castells N. (2021). Chromosomal microarray analysis in fetuses with central nervous system anomalies: an 8-year long observational study from a tertiary care university hospital. Prenat. Diagn. 41 (1), 123–135. 10.1002/pd.5829 32926442

[B26] Tang H. Van Pham H. Nguyen C. Nguyen H. Luu H. T. (2024). Comparative efficacy of cnv-sequencing and karyotyping in prenatal genetic diagnosis. Biomed. Res. Ther. 11 (7), 6622–6632. 10.15419/bmrat.v11i7.908

[B27] Verdoni A. Hu J. Surti U. Babcock M. Sheehan E. Clemens M. (2021). Reproductive outcomes in individuals with chromosomal reciprocal translocations. Genet. Med. 23 (9), 1753–1760. 10.1038/s41436-021-01195-w 33972719

[B28] Wang J. Chen L. Zhou C. Wang L. Xie H. Xiao Y. (2018). Prospective chromosome analysis of 3429 amniocentesis samples in China using copy number variation sequencing. Am. J. Obstet. Gynecol. 219 (3), 281–287. 10.1016/j.ajog.2018.05.030 29852155

[B29] Xu W. Deng C. Li W. Wang K. Tao J. Gao Y. (2020). National perinatal prevalence of selected major birth defects — China, 2010−2018. China CDC Wkly. 37 (2), 711–717. 10.46234/ccdcw2020.195

[B30] Xue J. Shen R. Xie M. Liu Y. Zhang Y. Gong L. (2021). 22q11.2 recurrent copy number variation-related syndrome: a retrospective analysis of our own microarray cohort and a systematic clinical overview of ClinGen curation. Transl. Pediatr. 10 (12), 3273–3281. 10.21037/tp-21-560 35070841 PMC8753460

[B31] Zhang H. Xu Z. Chen Q. Chen H. Ding X. Liu L. (2023). Comparison of the combined use of CNV-seq and karyotyping or QF-PCR in prenatal diagnosis: a retrospective study. Sci. Rep. 13 (1), 1862. 10.1038/s41598-023-29053-6 36725972 PMC9892513

[B32] Zhang S. Xu Y. Lu D. Fu D. Zhao Y. (2022). Combined use of karyotyping and copy number variation sequencing technology in prenatal diagnosis. PeerJ 10, e14400. 10.7717/peerj.14400 36523456 PMC9745786

[B33] Zhou X. Zeng X. Fang J. He J. Kuang H. Hua X. (2024). Comparison of total prevalence, perinatal prevalence, and livebirth prevalence of birth defects in Hunan Province, China, 2016-2020. Front. Public Health 12, 1297426. 10.3389/fpubh.2024.1297426 39324160 PMC11422065

